# Animal Research: Transparency and Obligations

**DOI:** 10.3390/nu16010020

**Published:** 2023-12-20

**Authors:** Maria Luz Fernandez, Lluis Serra-Majem

**Affiliations:** 1 Department of Nutritional Sciences, University of Connecticut, Storrs, CT 06269, USA; 2 Centro de Investigación Biomédica en Red Fisiopatología de la Obesidad y la Nutrición (CIBEROBN), Institute of Health Carlos III, 28029 Madrid, Spain; lluis.serra@ulpgc.es; 3 Research Institute of Biomedical and Health Sciences (IUIBS), University of Las Palmas de Gran Canaria, 35001 Las Palmas, Spain

As Editors-in-Chief of *Nutrients*—one of the largest nutrition journals in the world—we would like to comment on this subject because it will remain topical for as long as it exists.

Animal experimentation is a profoundly emotive and divisive subject. Traceable at least as far back as the work of the Ancient Greek anatomist Galen of Pergamum (129–ca. 216 C.E.), it could be claimed to have laid some of the key foundations of medical science as we know it today, and for at least the past two centuries, it has been the cause of passionate and often furious debate.

Our objective here is not to mount a defence of animal experimentation per se. We are Editors who work on behalf of an academic journal, and publishing houses do not engage in animal experiments.

All scientific publishing houses—including MDPI—exist to help advance science by facilitating the dissemination and discussion of new research findings. Recent criticisms levelled at *Nutrients* concerning the publication of a paper drawing on results obtained using experiments on pigs confuse the function of a neutral academic publishing platform, which MDPI is, with that of an academic research institution that conducts experiments for subsequent publication.

The insistence on this distinction is not to be confused with a washing of hands. *Nutrients* actively supports the long-established international regulations on animal experiments carried out by scientific institutions and pursues the enforcement of these. It is totally unacceptable and indeed libellous to publicly claim otherwise.

At the same time, we are cognizant of the fact that there may be cases where, at present, the use of animal experimentation may be the only means of testing the safety and efficacy of new therapeutic interventions that have the potential to combat disease, reduce suffering, improve quality of life, and even prevent mortality in human beings. We believe that in such cases, animal experimentation may be justified where there is no other means available to secure certainty about the safety and efficacy of potential new therapeutic approaches.

However, we all agree that animal experimentation should never be the preferred method of testing. 

Nor should it be regarded as a failsafe way of demonstrating drug safety and efficacy in and of itself. However, there may be instances where animal experimentation offers the only viable means of securing therapeutic benefits that will help alleviate forms of human suffering that are currently unaddressed. 

We would like to call on all institutions that carry out such tests to do everything in their power to adopt the necessary measures and controls so as to obviate the need for animal experimentation in the first place. 

The exponential growth of computing power over the past quarter-century has made the evolution of open access publishing houses such as MDPI possible. It has also led to the creation of many highly viable alternatives to animal experimentation. We invite researchers with an interest in this topic to submit their research findings, whether positive or negative, to relevant academic journals—ideally open access journals—so as to facilitate and accelerate the identification of alternative testing methodologies that might make animal experimentation a thing of the past. As a global scientific community, we have the potential to think our way towards a better future—and a moral obligation to do so.

